# Paralysis of Down-Gaze

**Published:** 1975-04

**Authors:** David G. Cogan

**Affiliations:** Howe Laboratory of Ophthalmology, Harvard University Medical School, Boston, Mass.


### Paralysis of Down-gaze

By

**David G. Cogan, M.D.,**

Howe Laboratory of Ophthalmology, Harvard University Medical School, Boston, Mass.

The substance of Dr. Cogan's paper appeared in the Archives of Ophthalmology, **91**, 192-199 (1974) and an abstract is therefore given below.

Paralysis of down-gaze is less frequent than that of up-gaze and therefore has had less attention paid to it. It is, however, occasionally the only presenting ocular sign of disease of the central nervous system. By down-glaze paralysis is meant impairment of conjugate eye movements, downward, either on command or in pursuit of a target and associated with proportionately little involvement of horizontal movement. The vestibular reflexes are intact and the eyes will move involuntarily when the head is thrust backward (the doll's head phenomenon).

The symptoms with down-gaze paralysis are disconcerting; there is often disturbance of gait and compensation is brought about by tilting the head forwards. It may also be associated with an inability to use bifocal spectacles.

There are four categories of down-gaze palsy: (1) reverse Parinaud's Syndrome; (2) down-gaze paralysis with choreo-athetosis; (3) down-gaze paralysis with parkinsonoid symptoms; and (4) miscellaneous cases.

1. Reverse Parinaud's Syndrome.

This abnormality is a paralysis of down-gaze associated with convergence paralysis and pupillary reflexia. The lesion is presumed to be in the mesencephalon.

2. Down-gaze paralysis with choreo-athetosis.

Choreo-athetosis is defined as involuntary, repetitive and sometimes continuous movements of the limbs, head, and trunk that occur with a variety of acquired and hereditary abnormalities having ill-defined lesions in the corpus striatum and brain stem. Clinical entities characterized by choreo-athetosis include Sydenham's chorea (usually rheumatic,) Huntingdon's chorea (hereditary), Wilson's disease, paralysis agitans, and congenital athetosis. Paralysis of up-gaze has been described with congenital athetosis (Churchill and Colfelt, 1963; Walsh and Hoyt, 1969), and I have previously emphasized the frequency with which down-gaze is impaired with Huntingdon's chorea (Cogan, 1956), but to my knowledge no series of these latter cases has been reported heretofore. Seven cases accorded with this group.

3. Down-gaze paralysis with parkinsonoid diseases.

Weakness of upward gaze is a frequent, though usually asymptomatic, finding in patients with Parkinson's disease. It may occasionally be associated with weakness of down-gaze but preferential paralysis of down-gaze paralysis must be infrequent with Parkinson's disease. On the other hand, selective paralysis of down-gaze is highly characteristic of the progressive supranuclear paralysis, as described by Steele *et al* (1964) and is one of the hallmarks of this entity. But the vestibulogenic movements are intact in both diseases producing the familiar controversive movements of the eyes with rotation of the body.

4. Miscellaneous cases.

Miscellaneous cases such as those very rare instances in which down-gaze paralysis occurs following strabismus surgery or pseudo-ophthalmaplegia.

**SUMMARY**

Classification of down-gaze paralysis is presented on the basis of 29 patients studied. It transpires from this that there is no one particular centre which can be responsible for this phenomenon.

**REFERENCES**

CHURCHILL, J. A. and Colfelt, R. H.: Etiologic factors in athetotic cerebral palsy. Arch. Neurol (Chicago) 9: 400-406. October (1963).

COGAN, D. G.: **Neurology of the Ocular Muscles.** Charles C. Thomas Company, Springfield, Illinois (1956).

STEELE, J. C., Richardson, J. C. and Olewski, J.: Progressive supranuclear palsy. Arch. Neurol. (Chicago) **10**: 333-359 April (1964).

WALSH, F. B. and Hoyt, W. F.: **Clinical Neuro-ophthalmology.** Williams and Wilkins Company, Baltimore (1969).

